# Multimodality image fusion in dose escalation studies of brain tumors

**DOI:** 10.1120/jacmp.v4i1.2545

**Published:** 2000-09-01

**Authors:** D. Rajasekar, N. R. Datta, R. K. Gupta, P. K. Pradhan, S. Ayyagari

**Affiliations:** ^1^ Department of Radiotherapy Sanjay Gandhi Postgraduate Institute of Medical Sciences Lucknow 226 014 India; ^2^ Radiodiagnosis and Imaging Sanjay Gandhi Postgraduate Institute of Medical Sciences Lucknow 226 014 India; ^3^ Department of Nuclear Medicine Sanjay Gandhi Postgraduate Institute of Medical Sciences Lucknow 226 014 India

**Keywords:** multimodality imaging, image fusion, brain tumor, 3D radiotherapy planning, dose escalation

## Abstract

This article examines the utility of integrating images from computed tomography (CT), magnetic resonance imaging (MRI), and single photon emission computed tomography (SPECT) for radiation treatment planning of brain tumors for dose escalation studies. The information obtained from these imaging modalities is complementary to each other and could provide anatomic (through CT and MRI) and metabolic (through SPECT) information of the target. This anato‐metabolic target localization could be expected to facilitate precise radiation therapy planning for brain tumors by delineating the boundary between the tumor, edema, and the normal brain parenchyma and identify the viable tumor nidus with greater degree of certainty. This could in turn lead to minimize dose to the normal tissue and permit dose escalation to the region of interest. The utility of these anato‐metabolic imaging modalities for defining the clinical target volumes along with planning target volumes for different phases of the radiation therapy is illustrated. © *2003 American College of Medical Physics.*

PACS number(s): 87.53.Tf, 87.57.Gg, 87.61.Pk

## INTRODUCTION

The aim of radiation therapy is to give a high dose of radiation to the target volume while minimizing the dose to the adjacent normal tissues. This is feasible only if the region of interest (i.e., target) can be clearly outlined from the surrounding healthy tissue. Recent technologies focus on improvement of radiation dose delivery. The development of intensity‐modulated radiation therapy (IMRT) has a high priority in academic and industrial communities because of its promise of delivering higher tumor dose, thereby increasing tumor control probability (TCP) with reduced normal tissue complication probability (NTCP). However, the value of high quality IMRT could be further enhanced with precise tumor localization.

The x‐ray computed tomography (CT) has been widely used in radiotherapy planning since it provides a much needed electron density map for the accurate dose calculations. Nevertheless, due to its inherent poor soft‐tissue contrast, accurate localization of the tumor in brain could be quite uncertain. Magnetic resonance imaging (MRI) with its better soft‐tissue contrast and ability to acquire images in any oblique sections make this modality appropriate for brain tumor localization. However, its inherent inability to accurately image complex bone/air regions and potential distortions from the scanner or by the patient could lead to inaccuracies in dose calculations if used alone in the radiotherapy planning process.^1^–^4^ These distortions could be reduced to a minimum by using a relatively small field‐of‐view (FOV), an increased receiver bandwidth, and a fast spin echo acquisition sequences.^5^,^6^ The recent improvements in software technology provide the capability of integrating imaging data from multiple sources with the potential for defining tumor volumes for radiotherapy treatment planning (RTP) with a greater degree of precision.

In certain cases, e.g., glioblastoma multiforme (GBM), a combination of MRI and CT may provide more accurate target and normal tissue delineation than either modality alone, which could be further useful for dose estimation by the treatment planning system (TPS). The patterns of failure following high‐dose 3D conformal RT for such tumor revealed a high order (89%) of recurrences within 80% isodose region.^7^,^8^ Escalating the dose to this viable tumor nidus, might increase survival for such cases by reducing the incidence of local tumor recurrence. Isotope scans, such as single photon emission computed tomography (SPECT) and positron emission tomography (PET), provide the unique imaging of metabolically active parts within the tumor. This information is vital for dose escalation to the viable tumor regions and also for monitoring the effectiveness of therapy. Thus, combining the information from CT, MRI, and SPECT/PET imaging studies could help radiation oncologists to define the target with a greater degree of certainty.^9^ This study has been undertaken to explore the utility of integrating the images from CT, MRI, and SPECT for 3D planning with escalated doses (biological conformation) of brain tumors.

## METHODS AND MATERIALS

**Figure 1:**
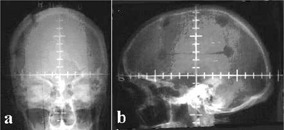
Simulator films showing reference lines in (a) antero‐posterior and (b) lateral projections.

The utility of the multimodality imaging for brain tumors is being illustrated in this case study. A patient with right temporal GBM referred for postoperative radiotherapy after partial excision of the tumor was considered for this study. The patient was immobilized using ORFIT thermoplastic immobilization system (ORFIT Industries, Belgium). Prior to obtaining scans from CT/MRI/SPECT scanners, the patient was taken up for preplanning simulation (SAT‐10, Shimadzu Corporation, Japan). Under fluoroscopic guidance, permanent reference lines in both antero‐posterior (AP) and lateral (LAT) projection were drawn on the immobilization cast, which were used for placing appropriate fiducial markers during multimodality imaging studies and tumor localization. In the AP view, the patient was positioned with the midline along the vertical grid line (*y* axis) and the horizontal grid line (*x* axis) along the superior border of the orbital foramen. In the LAT view, the vertical grid (*y* axis) was placed along the center of the auditory meatus (Fig. 1). Keeping this as isocenter, the vertical and horizontal grid lines were drawn on the immobilization cast in AP and lateral views.

### A. Imaging studies

*CT imaging.* With the patient immobilized properly on the couch angio‐catheters of appropriate lengths were fixed along the reference lines of the immobilization cast during the scan (Picker‐5500, Picker International Inc., USA). After contrast injection, axial cuts were taken from the top to the base of the skull with a field‐of‐view (FOV) 240 mm, a matrix size of 512×512, and a slice thickness of 10 mm. These were transferred to the treatment planning workstation (ASHA‐3D, indigenously developed in collaboration with CDAC, Pune and TSG Integrations, New Delhi, India). Our TPS utilizes bitmap images for planning and hence, tissue densities cannot be derived directly from bitmap images. Thus, we have used contrast‐enhanced CT (CECT) and the doses were calculated with standard inhomogeneity factors (equivalent tissue density for bone, 1.33; lung, 0.3; and air, 0.01). However, the Hounsfield values from DICOM files of plain CT with proper calibration, which forms direct correlation with the tissue density, should be preferable for treatment planning.

**Table I:** Clinical and planning target volumes for each phase of treatment.

Phase	Dose/fr.	Clinical target volume (CTV)	Planning target volume (PTV)
I	45 Gy/25 fr.	Volume of gross tumor along with surrounding edema $\left({\text{CTV}}_{1}\right)$	2.5 cm margin around ${\text{CTV}}_{1}\to {\text{PTV}}_{1}$
II	15 Gy/8 fr.	Gross tumor volume alone (${\text{CTV}}_{2}$)	2.0 cm margin around ${\text{CTV}}_{2}\to {\text{PTV}}_{2}$
III	6 Gy/3 fr.	Viable tumor volume obtained from SPECT (${\text{CTV}}_{3}$)	0.5 cm margin around ${\text{CTV}}_{3}\to {\text{PTV}}_{3}$

*MR imaging.* During MRI scanning (1.5 T, Magnetom, Siemens, Germany), oil filled tubes were placed on the immobilization cast along the reference lines as defined earlier. MRI scans were taken with the standard head coil. A custom‐made Perspex base plate, which could fit into the standard head coil but similar to the one used during simulation, was used for fixing the immobilization cast during MRI. Magnetic resonance (MR) images were obtained with spin‐echo (SE) sequence, which has least image distortions (<2mm within 240 mm diam).^10^ The conventional axial *T*2‐weighted (TR/TE 1,2/n=3000/12,80/1), plain and post‐contrast *T*1‐weighted (1012/14/2). MR images were obtained using a 256×256 matrix size, bandwidth of 65 Hz/pixel, FOV of 250 mm, and a slice thickness of 5 mm without a gap. Axial slices were taken from the top to the base of the skull. The MR images were stored in a standard format (ACER‐NEMA) and transferred to the TPS.

*SPECT imaging.* SPECT images were acquired by intravenous injection of ${}^{99}{}^{m}\text{Tc}$‐sestaMIBI (15 mCi). After 30 min, the patient was taken up for imaging with the dual head SPECT scanner (SOPHA, Sopha Medical Vision Inc., France). The patient was positioned with the immobilization cast with styrofoam neck‐rest, on a custom made flat‐wooden board for the SPECT study. Scalp vein tube filled with ${}^{99}{}^{m}\text{Tc}$ of specific activity around 5 *μ*Ci/ml, sealed and cut into desired lengths were used as reference markers. These were fixed along the three reference lines on the immobilization cast. SPECT scans were obtained in standard acquisition protocol (60 sec/projection, 32 projections, 64×64 matrix, FOV 500 mm, zoom=1). The filtered back‐projection method was used for reconstruction of images in axial sections (pixel size=7.8 mm) and transferred to the TPS.

### B. Target volume localization for different phases of treatment

Radiation therapy to the brain was delivered in three phases using shrinking fields. A total dose of 66 Gy was planned to be delivered in three phases: (a) Phase I, 45 Gy to gross tumor and surrounding edema, which perhaps contains microscopic disease; (b) Phase II, an additional 15 Gy to the gross tumor, and (c) Phase III, another 6 Gy to the viable part of the tumor. The details of the clinical target volumes (CTV) and the planning target volumes (PTV) as per ICRU 50^11^,^12^ for each of the three phases are outlined in Table I. Thus, three different CTVs, one for each phase, designated as ${\text{CTV}}_{1}$, ${\text{CTV}}_{2}$, and ${\text{CTV}}_{3}$ were obtained. Similarly, the corresponding PTVs for the three phases were nominated as ${\text{PTV}}_{1}$, ${\text{PTV}}_{2}$, and ${\text{PTV}}_{3}$. These volumes were drawn on each of the axial slices of the CT, MRI, and SPECT by the radiologists/nuclear medicine physician, in consultation with radiation oncologist. These imaging modalities were quantitatively analyzed using the TPS.

**Figure 2:**
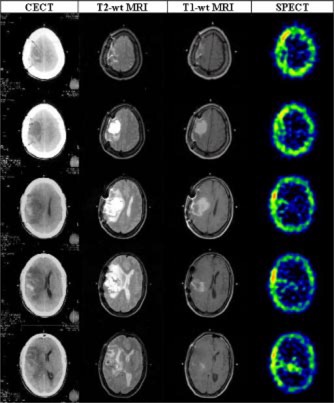
(Color) Corresponding axial sections of the CECT, *T*2‐weighted, *T*1‐weighted MR and SPECT images.

**Figure 3:**
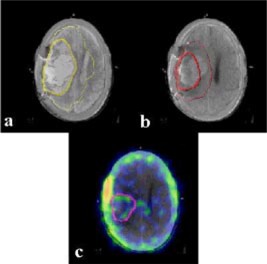
(Color) The axial section of CECT registered with corresponding images of MRI and SPECT. (a) CECT with *T*2 MRI for Phase I, (b) CECT with *T*1 MRI for Phase II, and (c) CECT with SPECT for Phase III.

*Image fusion and treatment planning.* The serial axial sections of the brain obtained by the CT, MRI (both *T*1 and *T*2), and SPECT were used to define the target volumes (Fig. 2). The clinical and planning target volumes were entered on each corresponding image slices for both CT and MRI data sets. Using the in‐house developed image fusion software based on rigid‐body transformation, registration was carried out with any two image sets viz. CT, MRI, and SPECT^1^,^13^,^14^ The 3D image sets have nine degrees of freedom, namely translation (*Tx,Ty,Tz*), rotation (*Rx,Ry,Rz*), and scaling (*Sx,Sy,Sz*) of the three principle axes (*x,y,z*). These nine parameters could be manipulated and visually verified in the fused image display to obtain the final fused image. The warping was avoided by choosing equal values for *Sx, Sy*, and *Sz.* Once the optimal matching achieved, by using the registration parameters, the match image set was re‐sliced at reference image locations by linear interpolation and a registered image set was created. These reference and registered image sets could be fused pixel to pixel to form a fused image set (Fig. 3) or could be used separately by the treatment planning system. This simple software tool helps in interactively register/fuse any two 3D image sets; the fiducial markers are merely a guide and the accuracy of the fusion depends on the operator.

Treatment planning was performed using 3D TPS with the registered images as mentioned previously. The beam portals were selected with the margins as given in Table I. In this illustrative case study, the Phase I treatment was planned with two parallel‐opposed lateral portals ($14\times 16{\;\text{cm}}^{2}$). The Phase II was given with lateral portal ($8\times 12{\;\text{cm}}^{2}$) along with wedged (45°W) AP, PA portals ($8\times 9{\;\text{cm}}^{2}$), while the Phase III treatment was planned with two oblique 45° wedged portals ($6\times 6{\;\text{cm}}^{2}$). The dose distribution was calculated with the standard inhomogeneity factors (viz. equivalent tissue density for bone, 1.33°; lung, 0.3°; and air, 0.01).

## RESULTS

**Figure 4:**
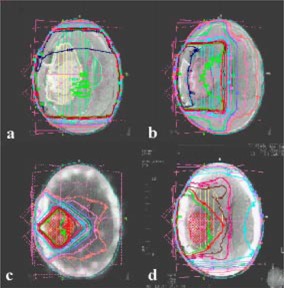
(Color) Isodose profiles of (a) Phase I, (b) Phase II, (c) Phase III, and (d) cumulative dose distribution of all three phases.

A glance at the serial sections of the corresponding axial cuts obtained by the CT, MRI, and SPECT images illustrates the differences in the information obtained from each of the imaging modalities (Fig. 2). This is expected due to the inherent physiological and physical basis of generation of these images. Thus in CT, there appears a considerable gray zone between the tumor, edema, and the surrounding normal brain parenchyma. On the contrary, the *T*2 MRI images have a better delineation of the edema with respect to the normal brain parenchyma. Although *T*1 MRI images could define the gross residual tumor with reasonable clarity, the viable part of the tumor was best visualized on the SPECT images. The resolution of the CT and MRI are far superior to that of the SPECT. Moreover, the different images generated by CT, MRI, and SPECT are complementary to each other for effective target localization and treatment planning. To plan the various phases of radiation therapy, CT formed the basis on which the corresponding images of MRI and SPECT were co‐registered: *T*2 MRI for Phase I [(Fig. 3a)], *T*1 MRI for Phase II [(Fig. 3b)], and SPECT for Phase III [(Fig. 3c)]. The isodose profiles of each of these phases and the cumulative dose distribution are shown in (Figs. 4a)–4(d).

In order to estimate the differences in the absolute volumes of CTVs and PTVs outlined in CT, MRI, and SPECT for each phase, the ICRU reference volumes, CTVs and PTVs, were marked on each slice and total volumes from CT, MRI, and SPECT imaging modalities were determined (Table II). Thus, considering the ICRU reference volumes (CTVs, PTVs) based on CT to be 100%, the MRI defined Phase I ${\text{CTV}}_{1}$ when compared to CT was larger by 13% and the ${\text{PTV}}_{1}$ showed an increase of 16%. This edema volume, believed to contain microscopic spread, would have been missed if CT alone was used for planning purposes in this patient. The MRI defined Phase II ${\text{CTV}}_{2}$ and ${\text{PTV}}_{2}$ volumes were smaller by 27% and 10%, respectively compared to CT volumes. This showed that MRI delineated the gross tumor volume better than CT Significantly, the viable tumor volume, obtained by SPECT study, showed 68% less volume compared to tumor volume obtained from CT and 60% less compared to MRI defined viable tumor volume. Probably this volume containing the viable cells perhaps would be responsible for tumor recurrence leading to treatment failures.^7^ Thus, it might be appropriate that the dose to this limited volume of viable tissue be escalated to achieve better tumor control. MRI and CT images tend to considerably overestimate the viable tumor volume compared to SPECT imaging modality as is evident.

## DISCUSSION

Malignant gliomas (anaplastic astrocytoma and glioblastoma multiforme) are the most frequent primary brain tumors encountered in the adult age group and represent a major cause of morbidity and mortality in neurological practice. The incorporation of CT/MRI information into the diagnosis of brain tumors provided an opportunity for earlier diagnosis as well as rigorous treatment and follow‐up.^2^,^8^ The use of contrast enhancement is indispensable in the evaluation of brain tumors and may be useful in identifying an isodense lesion from the surrounding normal parenchyma or hypo‐dense lesion hidden with an area of edema.^2^ Malignant gliomas characteristically have low signal intensity on *T*1 weighted and high signal intensity on *T*2 weighted images. Tumor cells extend at least as far as the margins of increased *T*2 signal. These tumors tend to infiltrate along white matter tracts and frequently involve and cross the corpus callosum. Enhancing tumors tend to have a higher histologic grade than nonenhancing tumors, but there are frequent exceptions and caution should be used when relating contrast enhancement to malignancy. Approximately 10% of glioblastomas do not enhance and a higher percentage of low‐grade gliomas show enhancement. Although the long‐term prognosis for patients with malignant brain tumors is poor, the choice of treatment protocol can have a major impact upon survival. The identification of patients who are likely to benefit from more aggressive treatment strategies is an important issue in patient management.

**Table II:** ICRU reference volumes (in CC) computed from CT, MRI, and SPECT images.

Target volumes	CT	MRI	SPECT
${\text{CTV}}_{1}$	254 (100%)	288 (+13%) ^b^	
${\text{PTV}}_{1}$	513 (100%)	293 (+16%) ^b^	
${\text{CTV}}_{2}$ ^a^	148 (100%)	108 (−27%) ^b^	
${\text{PTV}}_{2}$	317 (100%)	286 (−10%) ^b^	
${\text{CTV}}_{3}$ ^a^	148 (100%)	108 (−27%) ^b^	
${\text{PTV}}_{3}$	191 (100%)	153 (−20%) ^b^	61 (−68%) ^b^

^a^ CTV2 and CTV3 are identical for CT and MRI imaging.

^b^ The differences in the volumes are expressed with respect to CT, which is taken as 100%.

The detection of an active tumor and the functional assessment of the response to therapy are critical for patients with primary brain tumors such as malignant gliomas. These tumors are infiltrative in nature and the definition of tumor margins is extremely difficult using conventional imaging modalities. Although surgical resection is an important first line therapy, it is rare that all active tumors can be removed, and the rate of recurrence is relatively high. Adjuvant treatments such as radiation or chemotherapy are usually required to impede tumor growth.^7^ A direct, noninvasive measure of tissue function is mandatory for defining the extent of an active tumor, understanding the mechanisms of failure of different treatments, planning surgery or focal radiation therapy, and evaluating new therapeutic regimes. The most common site of relapse lies within the original tumor site or 2 to 4 cm of the margin. With a precise head‐fixation device, radiotherapy could be planned for a 2 to 3 cm margin outside the tumor border and surrounding edema.^4^,^12^,^15^

The conventional MRI sequences might enhance the necrotic regions as well as the active tumor, whereas the SPECT/PET images can differentiate the two.^9^,^16^ The single voxel proton magnetic resonance spectroscopy (MRS) and imaging, MRSI) has the potential to distinguish tumor relapse/necrosis and correlation of tumor grade.^17^,^18^,^19^,^20^ However, their use in the radiotherapy planning process is very limited due to their large voxel size (1 cc minimum) and time taken to acquire the spectrum of each voxel. Recent developments such as diffusion weighted imaging (DWI) also have the potential to image the functional status of the tumor and could be used in treatment planning process.^21^

The metabolic information about the tumor obtained from SPECT/PET is a valuable tool in dose escalation studies of brain tumors. ${}^{99}{}^{m}\text{Tc}$‐sesta‐methoxy‐isobutyl‐isonitrite (sestaMIBI), a cationic complex commonly employed in nuclear cardiology to visualize myocardial ischemia, can be used in brain imaging. Owing to the potential difference between the intercellular and intracellular compartments, the molecule can penetrate the cytoplasmic membrane and bind to mitochondria. For this reason, the tracer was proposed to detect brain tumor relapse and thereby distinguish between radiation or chemotherapy necrosis and viable lesions with high sensitivity and specificity.^9^ In the cerebral region, the sestaMIBI molecule distributes mainly in the scalp and in the choroid plexus. Pathological uptake is observed when there is a break in blood‐brain barrier due to the presence of a metabolically active neoplasm. This focal uptake by the active tumor can be registered with high‐resolution modalities such as CT or MRI and can be used in radiotherapy planning process. An additional dose to this focal uptake could be expected to result in improved survival. The recent developments of hybrid scanners, which inherently register SPECT/PET images with low contrast CT scans, are a valuable tool in radiotherapy planning.

The SPECT/PET can be expected to identify the presence of area of a viable region inside a tumor mass. However, both suffer from inherent limitations of poor spatial resolution. Hence, it becomes essential to incorporate both the anatomic images such as CT and MRI with the metabolic images SPECT/PET to generate anato‐metabolic images that could be used for radiation therapy planning. However, the absolute volume obtained from SPECT/PET imaging modalities may not be accurate due to their inherent poor spatial resolution (11.2 mm in our system), and the influence of various other factors, such as amount of activity injected, quality of the radiopharmaceutical used, time interval between injection and imaging, movement of the patient during imaging, type of filters used during the image reconstruction, display level, and its poor spatial resolution.

In general, integration of CT, MRI, and SPECT/PET in radiotherapy treatment planning has the ability to allow a more accurate definition of the target volume and thus better sparing of normal tissues. This could lead to an improved therapeutic ratio. This is evident in this illustrative case study, whereby the volumes of the various phases could be determined with reasonable certainty with multimodality imaging. However, the percentage change in volumes and the location and extent of brain tumor from different imaging modalities may vary significantly from patient to patient. The CT Vs MRI defined CTV for base of skull meningiomas^8^ was reported to be 17.6±10.8 cc Vs 19.6±14.2 cc. Volumetric analysis from another study for CNS tumors showed that the composite CT‐MRI gross tumor volume (GTV) was larger than CT defined GTV in all eight cases (mean increase of 72%).^22^

For Phase I target volume consisted of gross tumor and edema, which could be better delineated with a *T*2 MR image. In Phase II, the treatment portal was reduced to cover the gross tumor alone, and could be adequately depicted on a *T*1 MR image. The doses of 45 and 15 Gy to the Phase I and Phase II targets could be attained without enhancing the normal tissue damage. However, for any further dose escalation (6 Gy in our case), a precise location of viable cells within the gross tumor undergoing active metabolism becomes essential. SPECT/PET imaging scores over the other modalities in this aspect and hence can form an important adjunct to the CT/MRI.

An essential requirement of any attempt to fuse these images would be to minimize the spatial uncertainties associated with image acquisition. There are several sources of uncertainties in treatment planning and execution.^23^ The uncertainty associated with definition of tumor boundaries in the imaging data due to fundamental limitations of oncologic imaging (tumor/edema, microscopic extent of tumor) can be reduced by improved diagnostic imaging specificity and sensitivity. Improved patient immobilization could minimize the uncertainty in patient alignment during different imaging procedures. The accuracy of registration procedure could be verified by the alignment of rigid structures such as skull bone in brain tumors, which acts as a frame of reference. The uncertainty due to physiologic motion could be minimized by using a scanner with a faster data acquisition, or study the physiologic motion and determine anisotropic margins. Recent developments in software technology in image registration/fusion, hybrid cameras have contributed to reduce uncertainties in tumor localization substantially and hopefully could further pave the way for radiotherapy based on anatometabolic imaging.^22^,^24^

## CONCLUSIONS

This case study highlights the utility of multimodality imaging in the radiation therapy planning of brain tumors. One could be optimistic that with the feasibility of precise target localization in relation to the adjacent normal structures, radiation oncologist would be more confident in using dose escalation limited to the target volumes. This assumes further significance in the present era of conformal radiotherapy, intensity modulated radiotherapy, or even brachytherapy to deliver differentially higher doses to the target, based not only on the anatomic information of the disease but also on the metabolic information to differentiate necrotic or metabolically viable tumor tissue.
